# Relative Roles of Deterministic and Stochastic Processes in Driving the Vertical Distribution of Bacterial Communities in a Permafrost Core from the Qinghai-Tibet Plateau, China

**DOI:** 10.1371/journal.pone.0145747

**Published:** 2015-12-23

**Authors:** Weigang Hu, Qi Zhang, Tian Tian, Dingyao Li, Gang Cheng, Jing Mu, Qingbai Wu, Fujun Niu, James C. Stegen, Lizhe An, Huyuan Feng

**Affiliations:** 1 Ministry of Education Key Laboratory of Cell Activities and Stress Adaptations, School of Life Sciences, Lanzhou University, Lanzhou, China; 2 State Key Laboratory of Frozen Soil Engineering (SKLFSE), Cold and Arid Regions Environmental and Engineering Research Institute (CAREERI), Chinese Academy of Sciences, Lanzhou, China; 3 Biological Sciences Division, Pacific Northwest National Laboratory, Richland, United States of America; Institute of Tibetan Plateau Research, CHINA

## Abstract

Understanding the processes that influence the structure of biotic communities is one of the major ecological topics, and both stochastic and deterministic processes are expected to be at work simultaneously in most communities. Here, we investigated the vertical distribution patterns of bacterial communities in a 10-m-long soil core taken within permafrost of the Qinghai-Tibet Plateau. To get a better understanding of the forces that govern these patterns, we examined the diversity and structure of bacterial communities, and the change in community composition along the vertical distance (spatial turnover) from both taxonomic and phylogenetic perspectives. Measures of taxonomic and phylogenetic beta diversity revealed that bacterial community composition changed continuously along the soil core, and showed a vertical distance-decay relationship. Multiple stepwise regression analysis suggested that bacterial alpha diversity and phylogenetic structure were strongly correlated with soil conductivity and pH but weakly correlated with depth. There was evidence that deterministic and stochastic processes collectively drived bacterial vertically-structured pattern. Bacterial communities in five soil horizons (two originated from the active layer and three from permafrost) of the permafrost core were phylogenetically random, indicator of stochastic processes. However, we found a stronger effect of deterministic processes related to soil pH, conductivity, and organic carbon content that were structuring the bacterial communities. We therefore conclude that the vertical distribution of bacterial communities was governed primarily by deterministic ecological selection, although stochastic processes were also at work. Furthermore, the strong impact of environmental conditions (for example, soil physicochemical parameters and seasonal freeze-thaw cycles) on these communities underlines the sensitivity of permafrost microorganisms to climate change and potentially subsequent permafrost thaw.

## Introduction

Characterizing species diversity and its variation, or understanding the forces that structure ecological communities and their spatial patterns along environmental gradients is a central theme of ecological research, and both niche-related (deterministic) and neutral (stochastic) processes are generally thought to be important [1–4]. Niche-related processes [5] include selection imposed by the abiotic environment (environmental filtering) and biotic interactions (*e*.*g*., competitive exclusion, predation, facilitation, and mutualism), whereas neutral effects are related to chance or historical events (past evolutionary and ecological events that might influence present-day assemblages) [6], and include stochastic processes such as unpredictable disturbances, spatially restricted dispersal, mass effects and random fluctuations in population sizes [7]. It has been widely accepted that deterministic and stochastic processes jointly determine community assembly and many patterns previously assumed resulting from deterministic effects are also generated by stochasticity [2–4,7], which raises the question of how the relative contributions of these two sets of processes to the structure of ecological communities [8–12].

Taxonomic beta diversity (also referred to as taxonomic or species turnover), *i*.*e*., the change in community structure between sampling units along a spatial, temporal or environmental gradient [1], has provided important insights into the relative roles of deterministic and stochastic processes by relating the amount of turnover to variation in spatial distance and the abiotic environment [13–15]. While taxonomic beta diversity effectively quantifies the degree of similarity or dissimilarity in species composition between sites, it does not consider phylogenetic relatedness of species in the study system. Specifically, it can’t provide information about how deep in evolutionary time these species have been separated, because all species are treated equally in taxonomic beta diversity [16]. In recognition of the potential limitations of focusing solely on taxonomic beta diversity, community ecologists have recently extended this species-based metric to include a phylogenetic component of beta diversity (phylogenetic beta diversity or phylogenetic turnover) which should permit the inferences of relative roles of deterministic and stochastic processes that are more directly connected to ecological, historical, and evolutionary processes [11,16–19].

Deterministic and stochastic processes have long been investigated in animal and plant systems [20]. For example, Rominger et al. [8] and Gilbert and Lechowicz [21] have focused on assessing the relative importance of deterministic and stochastic processes in structuring grasshopper and understory plant communities, respectively. These processes have been explicitly studied in microbial communities in the past decade, and have been further integrated with microbial biogeography [3,6,20]. These studies provide overwhelming evidence of spatial patterns of microbial biodiversity, such as the distance-decay relationship and taxa-area relationship, which are similar to those of “macro”-organisms (*i*.*e*., plants and animals) [6,22]. Importantly, even though at small spatial scale, these patterns are also observed [23–25]. It should be noted that although previous microbial work has examined most of Earth’s major ecosystems [23], terrestrial cryoenvironments have received limited attention. This is a serious gap in our general knowledge of microbial ecology and diversity, given that the ecology of these cryoenvironments is largely microbial and vast regions of the planet remain at temperatures near or below freezing [26].

Permafrost, defined as ground (including soil, sediment, bedrock, and sand) that has been continuously frozen for at least two years, underlies about 25% of the Earth’s land area and is estimated to contain approximately 50% of the global soil carbon [27,28]. Permafrost occurs mostly in the northern reaches of North America and Eurasia (Alaska, Siberia, and Canada). It can be also found in the ice-free regions of Antarctica, Greenland, and surrounding Arctic and Antarctica as offshore permafrost, and in high mountains of Europe, both Americas, and western China as alpine permafrost [29]. Although permafrost environment is thought to be inhospitable, numerous and various microorganisms, including bacteria, archaea, phototrophic cyanobacteria and green algae, mycelial fungi, yeast, and protozoa, are present in it, and they play very important role in regulating biogeochemical processes, such as nutrient turnover and biomass production [30,31]. There has been increasing attention on the microbial ecology of permafrost recently because the reservoir of permafrost carbon may be susceptible to microbial decomposition as increasing global temperatures and possible subsequent permafrost thaw and result in greenhouse-gas (primarily CO_2_, CH_4_ and N_2_O) emissions [27,32–34]. Improved knowledge of factors that influence the composition and distribution of permafrost microbial communities is critical to advancing the microbial ecology of permafrost and predicting the potential consequences of climate change. Advances in characterizing microbial communities in permafrost commonly lead to inferences regarding whether community composition is significantly related to specific abiotic factors, such as nutrient availability [35], soil pH [36], moisture [37], conductivity [38] and profile depth [39]. Although microbial communities don’t seem to always vary with permafrost depth [40–42], some studies have indicated that microbial abundance, alpha diversity and metabolic activity show declines with depth across the transition from surface active layer soil to underlying permafrost soil, and community composition is significantly different between these two layers [38,43–45]. These results imply a high level of microbial turnover along depth or environmental gradients. However, the vertical distribution patterns of microorganisms in permafrost are still poorly understood, and the degree of β-diversity has not been strictly examined. In addition, the roles of deterministic and stochastic processes and their relative influences on vertical variation in permafrost microbial communities remain unexplored.

Here, we investigated vertical distribution patterns of bacterial communities and the processes that drive these patterns in a 10-m-long permafrost core from the Qinghai-Tibet Plateau, China. Bacterial community composition was analyzed using culture-independent techniques (cloning—restriction fragment length polymorphism analysis of PCR-amplified 16S rRNA gene fragment). We employed an integrated approach to characterize bacterial diversity from both ecological and evolutionary perspectives. Therefore, in addition to evaluating patterns of taxonomic beta diversity along the soil core, we included several phylogenetic measures, that is, phylogenetic diversity, community structure, and turnover of bacterial communities. Especially, coupling the phylogenetic community structure and turnover with randomly generated null models can contribute to illuminate the relative roles of deterministic and stachostic processes in structuring these communities [16,46,47]. The major objectives of this work were to address the following questions: (1) what are the vertical distribution patterns of bacteria through the permafrost core profile with regard to taxonomic and phylogenetic beta diversity? (2) Which ecological processes (deterministic or stochastic processes) is more important to the vertically-structured shifts in bacterial communities?

## Materials and Methods

### Ethics statement

The permafrost core was collected in the state-owned land which is open for scientific research. No specified permissions are required for this sampling site, which is not natural reserve and did not involve endangered or protected species.

### Study area and sampling

The study area was situated in the Kunlun Mountain Pass (N35°39′28.6″, E94°03′17.3″), Qinghai-Tibet Plateau, which is a typical alpine tundra region with an altitude of about 4780 m above sea level. The annual mean air temperature and precipitation at this site are -5.0 to -7.0°C and 400 mm, respectively. The depth of the active layer at the time of sampling is approximately 260 cm. The sampling site is composed of alpine meadow, and the dominant vegetation was *Oxytropis glacialis*, *Stipa purpurea* and *Kobresia alpine*. Sampling was carried out in August 2010; a 10-m-long soil core was taken within permafrost using the coring equipment and protocols described by Hu and colleagues [38]. Core was split at the different depths using a sterilized chisel, and soil samples were collected from seven depth intervals representing the upper active layer at 50 ± 5 cm; 100 ± 5 cm; 150 ± 5 cm; 175 ± 5 cm; 200 ± 5 cm; 225 ± 5 cm and 250 ± 5 cm, and at nine depth intervals within the underlying permafrost: 275 ± 5 cm; 300 ± 5 cm; 400 ± 5 cm; 500 ± 5 cm; 600 ± 5 cm; 700 ± 5 cm; 775 ± 5 cm; 900 ± 5 cm and 975 ± 5 cm. Three replicate samples (~50 g each) were subsampled from the inner portion of each interval with sterilized scalpels and tweezers. A total of forty-eight soil subsamples (16 × 3) were immediately put into aseptic aluminium tins, sealed and maintained at -20°C until processing in the lab.

### Soil physical and chemical analyses

Physicochemical analyses of soil pH, conductivity, water content, and concentrations of soil organic carbon (SOC) and total nitrogen (TN) were performed as previously described [38]. In brief, the soil moisture was determined as the differences in mass of fresh soil dried at 105°C for 24 h. Air-dried soils were passed through a 100-mesh screen, then concentrations of SOC and TN were determined using the CHNS-analyzer system (Elementar Vario EL, Elementar Analysensysteme GmbH, Hanau, Germany) with the burning method at 450 and 1250°C, respectively. Soil pH was measured using 1M KCl (5 g soil in a 25mL solution), and soil conductivity was determined in 1:2 soil/deionized water slurry.

### Soil DNA extraction and amplification

For each soil subsample, total community DNA was extracted using the modified method described previously [38]. Prior to DNA extraction, soil samples of five grams were thoroughly ground with liquid nitrogen in a pre-chilled sterile mortar and subjected to successive washes by vortexing with buffers differing in EDTA concentrations. Samples were then centrifuged for 3 min (3000 × *g*). The soil pellets were mixed with hexadecyltrimethylammonium bromide (CTAB) extraction buffer (0.1 M Tris-HCl, 0.1 M sodium EDTA, 0.1 M sodium phosphate, 1.5 M NaCl and 1% CTAB) and proteinase K (10 mg/mL) and shaken at 225 rpm for 30 min. After shaking treatment, 20% sodium dodecyl sulfate (SDS) was added, and the mixtures were incubated at 65°C for 2 h. The supernatants were collected by centrifuging and extracted with chloroform-isoamyl alcohol (24:1). The aqueous phase was precipitated with isopropanol, and pellets of crude nucleic acids were washed with cold 70% ethanol and resuspended in sterile deionized water. Extractions from three replicates at each sampling depth were pooled at this step and analyzed as one sample in the subsequent analyses (resulting in sixteen pooled DNA samples). Pooled community DNA was purified using the Universal DNA Purification Kit (Tiangen Biotech, China) according to the manufacturer’s instructions. A negative parallel control (deionized H_2_O in place of soil or DNA) underwent identical procedures during extraction and purification processes to evaluate the potential for contaminations.

Bacterial 16S rRNA genes were amplified from the purified community DNA and negative parallel control by PCR using the universal primer pair 27F (5′-AGAGTTTGATCCTGGCTCAG-3′) and 1492R (5′-TACGGTTACCTTGTTACGACTT-3′). Amplification reactions were performed in a total volume of 25 μL containing 0.5 μM of each primer and 3 μL of template DNA using a *Taq* PCR Kit (New England Biolabs, MA, USA) with the following thermocycling conditions: an initial denaturation step of 5 min at 94°C and then subjected to 35 amplification cycles of 1 min denaturation at 94°C, 1 min annealing at 58°C, followed by 72°C for 1 min 30 s and a final extension of 72°C for 10 min. To mitigate individual PCR reaction biases, each amplification was performed in three replicates and pooled together. All PCR reactions were carried out on a thermal cycler (Applied Biosystems GeneAmp^®^ PCR System 2700). The presence or absence of PCR products was determined on a 1.0% (w/v) agarose gel with ethidium bromide staining.

### Cloning, restriction fragment length polymorphism (RFLP) typing and sequencing

Ligation and transformation of amplified 16S rRNA genes were performed as previously described [38], ultimately resulting in 16 bacterial clone libraries. For each clone library, 450 putative positive transformants were picked randomly and immersed in 30 μL of deionized H_2_O, and subjected to three cycles of freezing and thawing for the preparation of plasmid templates. Cloned 16S rRNA genes were re-amplified using the primer pair T7 and SP6. PCR reactions were performed in a 20 μL mixture with 0.4 μM of each primer and 1 μL of template DNA using a *Taq* PCR Kit (Tiangen Biotech, China) with the same PCR conditions as amplification of community DNA, with the exception that only 30 cycles were performed. Restriction fragment length polymorphism (RFLP) analysis was used to distinguish and classify cloned 16S rRNA gene sequences. A total of 6753 positive PCR products were restricted using the enzymes *Hin*fI and *Csp*6 (Fermentas, Vilnius, Lithuania) at 37°C for 3.5h. Restriction digests (10μL) were examined on 3.0% (w/v) agarose gels, and unique restriction patterns were identified visually. Representatives of each restriction pattern were chosen for sequencing using the vector primer pair T7 and SP6 by the Major Biotech Co., Ltd (Shanghai, China).

### Molecular analyses

All DNA sequences were edited and assembled using the CONTIGEXPRESS module of VECTOR NTI Suite 6.0 (InforMax Inc., MD). Sequences were checked for chimeras using the online CHIMERA CHECK program on the RDP II database (http://rdp.cme.msu.edu/index.jsp). Identified chimeric sequences were removed from the dataset before further analysis, ultimately resulting in a total of 373 bacterial sequences. The remaining 373 sequences of bacteria were submitted to the GenBank database under the accession numbers KF494429-KF494801. Phylogenetic classifications of non-chimeric sequences were carried out using the EzTaxon-e online database [48]. All bacterial sequences were then multiple aligned using the CLUSTAL W program [49], and clustered to species-level groups using 97% pairwise identity with the furthest neighbor algorithm in the MOTHUR program [50]. Each species-level group was regarded as a bacterial phylotype (OTU). Representative sequences from each bacterial phylotype were used to construct a phylogenetic tree using General Time Reversible and gamma substitution models with the RaxML algorithm implemented in TOPALi package ver. 2.5 [51]. A ‘newick’ format of the *Bacteria* phylogenetic tree was saved to use for subsequent phylogeny-related analyses.

### Statistical analyses

The matrix of bacterial community composition was calculated using the clone numbers of each phylotype in each soil sample. The raw data of soil physicochemical characteristics that were measured on three replicate subsamples were pooled and calculated, using the means to represent the status of each variable. All statistical analyses were carried out using SPSS 13.0 (SPSS Inc., Chicago, IL, USA) and R (version 3.0.2; http://www.r-project.org). Before analysis, all data were tested for normality; all the soil physicochemical variables met the normality distribution; further, these variables were standardized at a mean of 0 and a standard deviation of 1. Raw community data for bacteria was Hellinger-transformed in order to make sure the contribution of abundant and rare phylotypes were equally important for the resultant matrix.

Chao1-richness was computed to estimate the community diversity using the MOTHUR program [50]. Chao1-richness is a non-parametric estimator of phylotype richness that is calculated as Chao1-richness = *S*_obs_ + [*a* ^2^/(2 × *b*)], where *S*_obs_ is the observed number of phylotypes, and *a* or *b* is the number of phylotypes with only one sequence or only two sequences. Furthermore, Faith’s PD [52] was calculated to estimate phylogenetic community diversity, which was quantified as the sum of the branch length in a phylogeny that connects all species within the community and the root.

To characterize phylogenetic community structure within each sample, we calculated the mean nearest taxon distance (MNTD) [47]. A randomly generated null distribution of MNTD was computed by the “taxa.labels” null model with 999 iterations using the function ‘ses.mntd’ from the library “picante” [53] of R package. To assess vertical structure in the degree of non-random phylogenetic community structure, nearest taxon index (NTI) was quantified as the number of standard deviations that the observed MNTD was from the mean of the MNTD null distribution [47]. Observed NTI values smaller than 50 or larger than 950 of the randomizations were considered significantly structured (*P* < 0.05 or *P* > 0.95). For a single community, a significantly positive or negative NTI value indicated that co-occurring species were more closely or distantly related than expected by chance. Based on the hypothesis that closely related taxa were more ecologically or functionally similar (phylogenetic niche conservatism), the obtained NTI measure can be used to infer ecologically similar (phylogenetic clustering) or ecologically dissimilar (phylogenetic overdispersion) taxa within a given community [47]. A mean NTI taken across all communities that was significantly different from the expected value of zero was interpreted as indicating an average trend towards clustering (NTI > 0) or overdispersion (NTI < 0) [54]. To correlate the depth and environmental variables with the observed biodiversity patterns, a step-wise multiple regression analysis with forward model selection was performed for each biodiversity measurement. The model with the lowest Akaike information criterion (AIC) was selected.

To examine the beta diversity patterns of bacterial communities, the Bray-Curtis metric was computed to describe the dissimilarity in species community composition (taxonomic beta diversity) between all pairwise comparisons of bacterial communities [46]. Phylogenetic beta diversity between a given pair of samples was quantified using beta mean nearest taxon distance (betaMNTD) and the beta nearest taxon index (betaNTI), which is the between-community analogs of MNTD and NTI, respectively [54]. BetaNTI measured the difference between observed betaMNTD and mean null betaMNTD for a given pair of communities in units of standard deviations [54]. Variation in beta diversity among bacterial communities was examined using a distance-based approach [6,13,14]. That is, the variations in beta diversity were correlated with changes in vertical distance or environmental distance. The resulting vertical distance-decay relationships (which measure how dissimilarities decay with increasing distance between pairwise soil horizons) [24,55] were analyzed using Generalized Linear Models (GLM), and significance levels were determined using Mantel tests (Pearson’s correlation) with 9999 permutations. Environmental distance was measured as Euclidean distance using all the standardized environmental variables. To facilitate comparisons with previous studies, we also calculated the distance-decay slope using the Jaccard metric for taxonomic beta diversity. Furthermore, we constructed multiple regression models to evaluate the relationship between beta diversity metrics and vertical or measured environmental distance after controlling for measured environmental distance or vertical distance, and significance was assessed using partial Mantel tests with 9999 permutations.

To estimate the relative importance of deterministic and stochastic processes on bacterial distributions, variation partitioning was performed for taxonomic and phylogenetic beta diversity. We partitioned beta diversity into spatial and environmental components using both the distance-based approach and the raw data approach as previously described [13,15,24], as they may give additional insights into the data, reflecting different aspects of beta diversity [14]. Further, in order to elucidate the relationship between environmental variables and community dissimilarities, we used non-metric multidimensional scaling (NMDS) ordination technique. Measured environmental variables were fitted as vectors onto the ordination plots, and the significance of each variable was assessed. These analyses were implemented in R packages “picante” [53], “vegan” [56] and “car” [57].

## Results

### Physical and chemical characterization of permafrost

**Fig 1:**
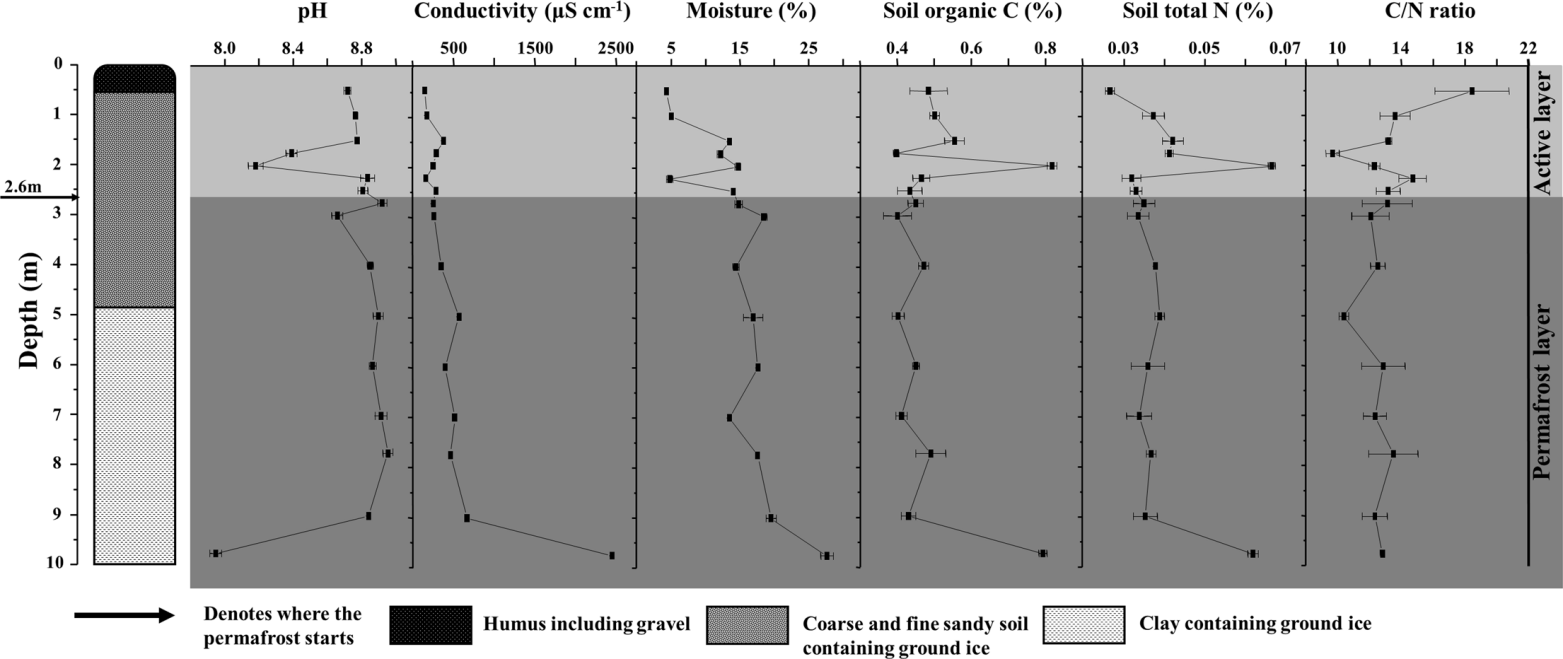
Schematic diagram of the permafrost core and the associated physiochemical characteristics of different-depth samples. Error bars represent means ± the standard error (n = 3).

Our soil samples were collected from different depth intervals within a 10-m-long permafrost core, which progresses from surface active layer soil into a layer of permafrost soil. The stratigraphic profile and soil physicochemical characteristics across depths are shown in Fig 1. The soils of the surface layer (approximately surface to 0.6 m in depth) were dominated by humus soil, with variable amounts of gravel. In the range 0.6–4.8 m, the zone was composed of coarse and fine sandy soil, containing ground ice. Below 4.8 m, the soils were typically grey and dark grey in colour, with the exception for a black layer at 9.6–10.0 m. This zone consisted of clay and also contained ground ice. The soils throughout the permafrost core profile were alkaline and had low SOC and TN contents. The soil pH ranged from 7.88 to 9.00 (mean = 8.71, SE = 0.04, n = 48). The concentrations of SOC and TN varied from 0.36 to 0.84% (dry weight) (mean = 0.50%; SE = 0.02; n = 48) and 0.025 to 0.068% (dry weight) (mean = 0.039%; SE = 0.002; n = 48), respectively. Pearson correlation analyses showed that soil moisture (*r* = 0.769, *P* < 0.001) and conductivity (*r* = 0.696, *P* = 0.003) increase significantly with depth. No observable correlations were found between the other variables and depth.

The soil characteristics at the very bottom of the permafrost core (9.75 m) were completely different from those of the other soil horizons (Fig 1). This horizon contained higher conductivity, SOC and TN contents, and lower soil pH because it primarily consisted of organic soils rather than mineral soils which composed the other horizons.

### Analyses of bacterial 16S rRNA gene clone libraries

Nothing was recovered from the negative parallel controls, suggesting that contamination was not introduced during extraction and purification processes. To yield enough DNA for PCR amplification and minimize individual extraction biases, DNA extractions from three replicate subsamples were combined for each sampling depth and used as one sample for the construction of clone library (also see Materials and Methods). A total of 373 bacterial sequences (82.9% of raw sequences), based on 6753 bacterial clones, were obtained in this study. These sequences could be delimited into 191 phylotypes with sequence identity ≥ 97% (S1 Fig). Of these phylotypes, 139 corresponded to unique sequence and 52 to clusters of similar sequences.

These phylotypes belonged to 17 phyla and 52 orders (S1 Fig). The dominant phyla were *Alphaproteobacteria* and *Actinobacteria*, which showed the highest diversity, consisting of 46 phylotypes, respectively. Sequences related to *Betaproteobacteria*, *Gammaproteobacteria*, *Firmicutes*, *Acidobacteria* and *Bacteroidetes* were also frequently detected. In addition, phylotypes of *Deltaproteobacteria*, *Gemmatimonadetes*, *Planctomycetes*, *Verrucomicrobia*, *Saccharibacteria* (former candidate division TM7), *Armatimonadetes* (former candidate division OP10), *Cyanobacteria*, *Chloroflexi*, and *Nitrospirae* were identified at relatively low diversity, as well as members of an unclassified phylum (S1 Fig).

### Vertical distance-decay relationships, phylogenetic structure, and variation partitioning of bacterial community

**Fig 2:**
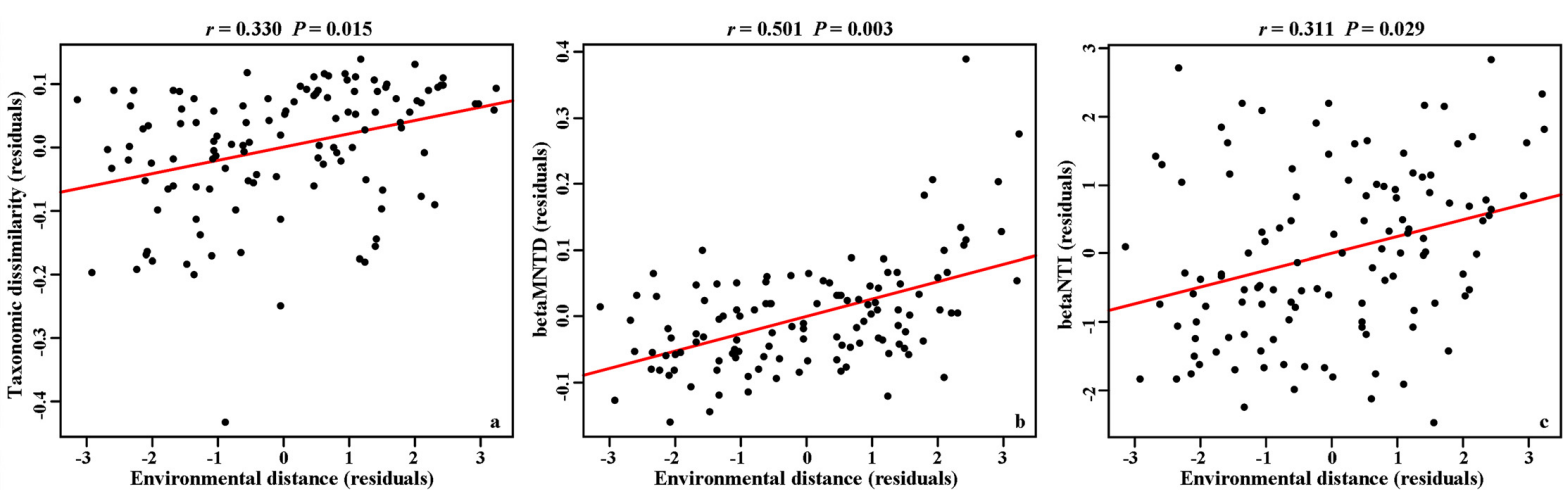
Partial correlation between environmental heterogeneity (environmental distance) and taxonomic dissimilarity (a); and betaMNTD (b); and betaNTI (c). Residuals of the x and y variables are plotted in order to account for the effects of vertical distance and spatial autocorrelation. Solid lines represent linear regressions and the significance levels are determined by partail Mantel tests (9999 permutations).

The taxonomic dissimilarity and betaMNTD significantly increased with spatial distance and environmental distance which showed obvious vertical distance-decay relationships for the whole core (S2 Fig). The slope of the distance-decay relationship based on the Jaccard metric for taxonomic beta diversity was -0.0132 In(Jaccard) per m of distance. According to the partial mantel tests, the pure effect of spatial distance was not significant for all three beta diversity metrics after controlling for environmental distance (*P* > 0.200 for all), while the pure effect of environmental distance was significant for all metrics after controlling for spatial distance (*P* < 0.030 for all; Fig 2).

**Fig 3:**
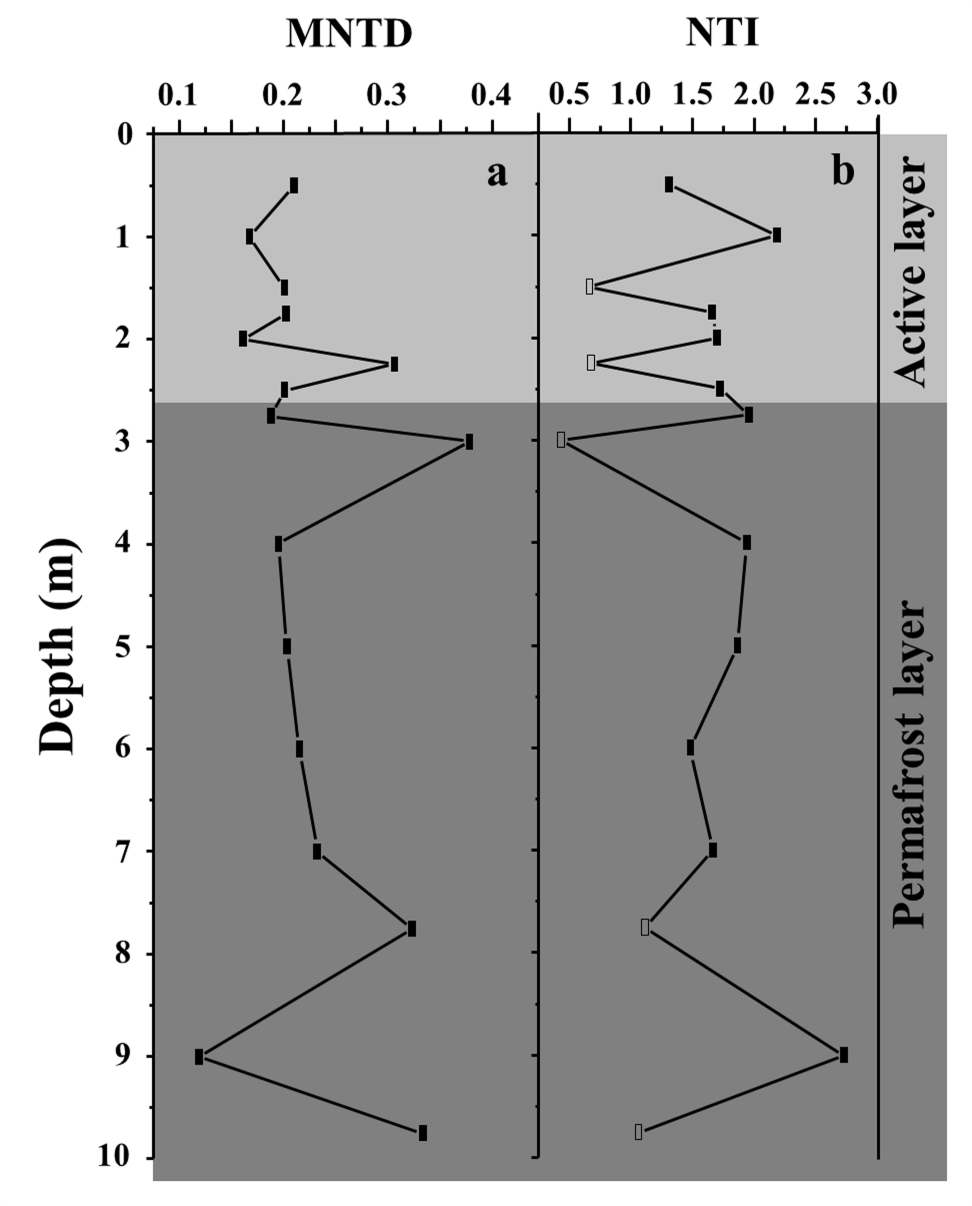
Mean nearest taxon distance (MNTD; a) and nearest taxon index (NTI; b) of different-depth bacterial communities. Observed community phylogenetic structures unlikely to arise by chance (*P* < 0.05 or *P* > 0.95) are depicted by solid symbols (b).

The analysis of phylogenetic structure revealed the phylogenetic patterns of bacterial communities through the permafrost core profile. The MNTD of 11 bacterial communities was significantly different from the null communities (significant values of NTI), whereas it was not significantly different from the null communities (non-significant values of NTI) in the other five communities, of which two (at depths of 150 and 225 cm) originated from the active layer and three (at depths of 300, 775, and 975 cm) from permafrost (Fig 3). All the NTI was greater than zero and the mean value of NTI (1.51) across all bacterial communities was significantly higher than the expected value of zero (*P* < 0.001; two-tailed *T* test at 95% confidence level). These results suggest that bacterial communities in 11 soil horizons were phylogenetically clustered and the other five communities were phylogenetically random, whereas there was an average trend towards clustering across all communities.

**Fig 4:**
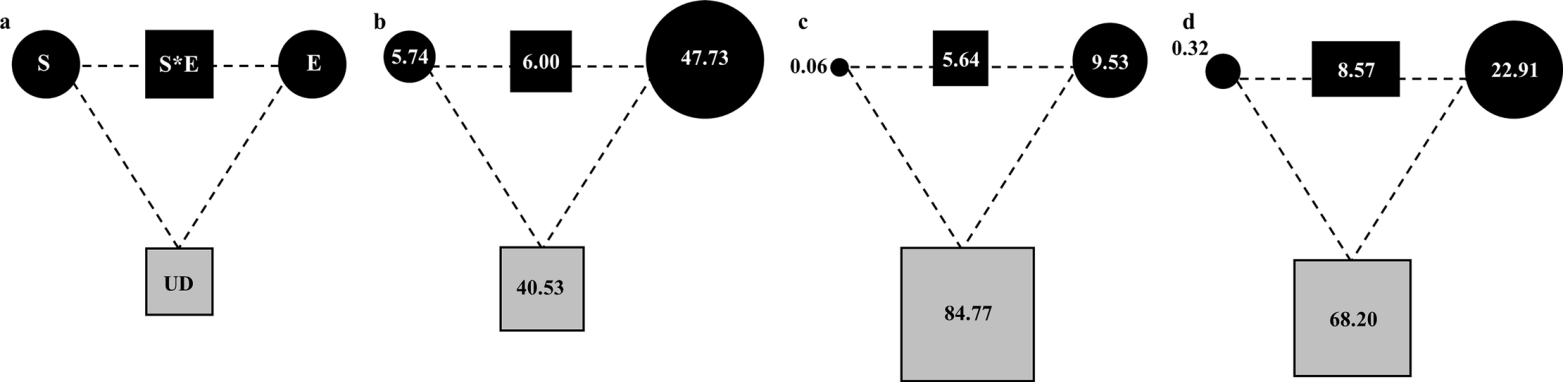
The proportion of variance in community composition explained by environmental components (E) and spatial components (S). (a) General outline. Each diagram represents variation in a given beta diversity metric partitioned into the relative effects of each component or combination of components. The edges of the triangle depict the variation explained by each component alone (*i*.*e*. when removing the variation because of other components). Percentage of variation explained by the vertically-structured environment is indicated by S*E. UD represents the variation unexplained; (b) abundance-based partition; (c) and (d) distance-based partition for taxonomic dissimilarity and betaMNTD, respectively.

Analysis of variation partitioning between spatial and environmental components (Fig 4) was used to tease apart the relative influences of deterministic and stochastic factors on bacterial distributions and beta diversity. When the abundance-based method was used (Fig 4B), the pure effect of environment accounted for larger parts of the variation in bacterial community composition (47.73%; pseudo-F = 2.12, *P* = 0.006) than the pure effect of space (depth) (5.74%; pseudo-F = 2.86, *P* = 0.015). When the distance-based method was employed, variation partitioning showed that the pure environmental distance explained 9.53% and 22.91% of the variation in taxonomic dissimilarity and betaMNTD, respectively, while the pure spatial distances explained less than 0.32% for the both metrics (Fig 4C and 4D). Furthermore, the fraction that was left unexplained was high, especially for taxonomic beta diversity (84.77%; Fig 4C).

### Relationships between diversity measurements and potential explanatory variables

The correlations between bacterial biodiversity and the environmental variables or depth were determined by step-wise multiple regressions. Environmental variables and depth explained a substantial fraction of variation in biodiversity (all *r* > 0.753; Table 1). The soil conductivity showed the strongest correlations with Chao1-richness and Faith’s PD. The soil pH was the most important correlate for MNTD and NTI. The depth was only relatively weak correlated with Chao1-richness and Faith’s PD.

**Table 1:** Relationships between the bacterial diversity and potential explanatory variables that were modelled using step-wise multiple regressions.

	*r*	Explanatory variables		
Chao1		Conductivity^***^	Depth^*^	
	0.982	-0.814^a^	-0.167	
Faith’s PD		Conductivity^***^	pH^**^	Depth^*^
	0.968	-0.898	0.553	-0.175
NTI		pH^*^	Conductivity^*^	
	0.753	1.983	-1.218	
MNTD		pH^***^		
	0.874	0.980		

The best models were identified using Akaike’s information criterion (AIC).

Chao1, Chao1-richness; Faith’s PD, Faith’s phylogenetic diversity; NTI, nearest taxon index; MNTD, mean nearest taxon distance.

^***^ *P* < 0.001

^**^ *P* < 0.01

^*^ *P* < 0.05.

^a^ Standardized partial regression coefficients.

**Fig 5:**
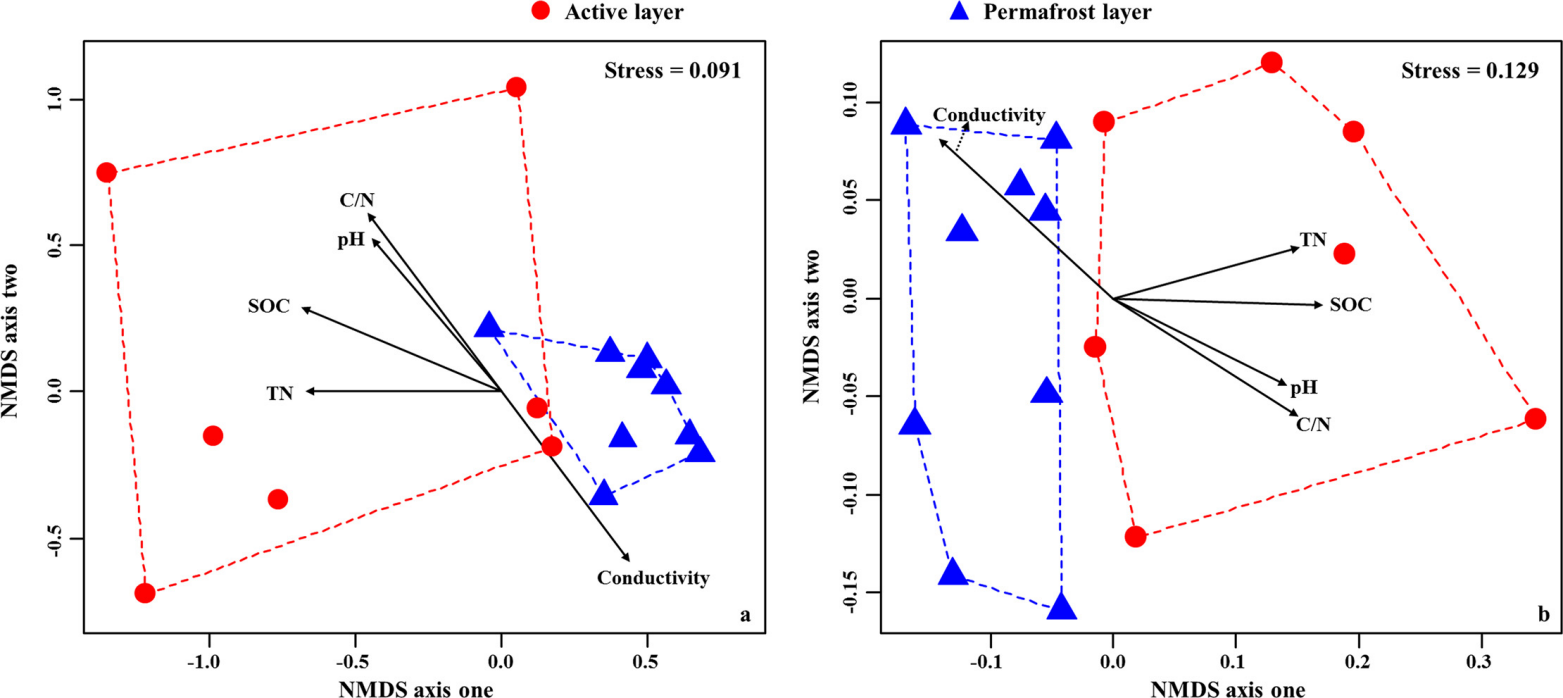
Non-metric multidimensional scaling (NMDS) plots of bacterial communities based on taxonomic dissimilarity (a) and betaMNTD (b) matrix, respectively. The stress value reflects how well the ordination summarizes the observed distances among samples (lower than 20% can be ecologically interpretable and useful). Soil variables were fitted as vectors onto each ordination plot, and significant vectors at 95% confidence level (*P* ≤ 0.05) were displayed.

The dissimilarity in species community composition of bacteria was significantly related with conductivity, C/N ratio, SOC, pH and TN (*P* < 0.05 for all); of which, the soil conductivity (*r* = 0.885, *P* = 0.002) was the most significant variable (Fig 5A). Similarly, these variables were strongly correlated with phylogenetic dissimilarity (*P* < 0.05 for all; Fig 5B), and the SOC (*r* = 0.807, *P* = 0.002) was the most relevant variable, followed by the soil conductivity (*r* = 0.770, *P* = 0.004).

## Discussion

Recent studies on an extensive range of different permafrost ecosystems have showed the presence of diverse forms of microorganisms [27,30,58]. And also, several reports have demonstrated the ecological drivers governing the microbial assemblage in such unique environment along a latitudinal [59] or elevational [60] gradient. Here we aimed to examine the vertical distribution of bacterial communities along a permafrost core in the context of both taxonomic and phylogenetic patterns, and further to assess the relative roles of deterministic and stochastic processes in structuring these communities. Although the number of samples used in our study prevents inference of the results to the wider Qinghai-Tibet Plateau permafrost environments, our data do provide evidence that bacterial communities are not randomly distributed through the permafrost core profile and their assembly is determined by ecological processes.

In the present study, the predominant bacterial phyla in the alkaline permafrost soils of the Kunlun Mountain Pass were *Proteobacteria*, *Actinobacteria*, *Firmicutes*, *Acidobacteria*, and *Bacteroidetes*. This is in agreement with some other studies [61–63] that were conducted in various permafrost regions in China, such as the Qilian Mountains, the Tianshan Mountains, and the Da and Xiao Xing’an Mountains. These results are also generally agree with the community composition reported from Arctic soils [27], suggesting these bacterial groups are well adapted to the extreme conditions of permafrost habitats. The dominance of *Actinobacteria* in permafrost environments could be largely attributed to their metabolic activity and DNA repair mechanism at low temperature [64]. *Firmicutes* are known to form endospores for resistance against long-term exposure to low temperature, desiccation and limited nutrient availability [44]. Bacteria belonging to the phylum *Acidobacteria* were reported to be abundant in permafrost potentially because of their oligotrophic attributes [65]. Several recent studies have showed that permafrost soils are dominated by representatives of uncharacterized bacterial phyla. For example, permafrost bacterial communities in a littoral wetland of Lake Namco, Qinghai-Tibet Plateau revealed different community composition which was mainly comprised of the sequences related to *Actinobacteria*, *Proteobacteria*, and *Chloroflexi* [66]. Because the members of *Chloroflexi* are adapted to survive in water-saturated soils, the high number of *Chloroflexi* in permafrost wetland of Lake Namco can be due to almost saturated water content in this area [66]. In Antarctic terrestrial ecosystem, in addition to the bacterial phyla mentioned above, sequences related to *Deinococuss*-*Thermus* and *Cyanobacteria* were often found with high levels [67]. Members of the phylum *Deinococuss*-*Thermus* are known for their ability in resisting to both low water availability and constant background radiation [68] to ensure their survival in extreme Antarctic soils. The cyanobacteria are generally thought to act as the primary producers of carbon and nitrogen in Antarctic soil system, in where environmental harshness precludes the survival of higher eukaryotic phototrophs [67]. Collectively, these results demonstrate that permafrost from different geographical locations could share a core set of microorganisms, however, some compositional differences are also observed. These differences may reflect the unique and extreme conditions of the permafrost environments [27].

Across the soil core, both species compositional dissimilarity and betaMNTD increased significantly with increasing spatial distance. These distance-decay relationships indicated that community composition changed continuously with increasing depth, from the active layer into permafrost. Previous studies have also found significant distance-decay relationships in microbial communities [69] and suggested that turnover rates in both taxonomic and phylogenetic community composition are obviously high for bacteria in the shallow terrestrial subsurface environments [23,24]. To compare with previous studies, we computed distance-decay slope using the Jaccard metric for taxonomic beta diversity. The resulting slope [-0.0132 In(Jaccard) per m of distance for the whole core] was much lower than the reported slope for bacteria in the shallow terrestrial subsurface (-0.304) [24] but was much higher than for tropical trees [70]. The relatively shallow slope—with respect to other bacterial communities—found here for taxonomic beta diversity was due in part to relatively high compositional dissimilarity between the most closely located sample pairs. It would be interesting to include community comparisons across shorter spatial distances to evaluate whether a steeper distance-decay slope would emerge due to greater compositional similarity across short distances. In contrast to taxonomic distance-decay, the slope of the phylogenetic distance-decay relationship (using betaMNTD) was 0.010 per m. This slope is similar to previous observations of high phylogenetic turnover rates for bacteria in terrestrial subsurface sediments from Kusai Lake (0.025 per m) or Lugu Lake (0.006 per m), and much larger than slopes from other habitat types [23]. Our distance-decay results collectively show strong vertical structure of bacterial communities along depth profile sampled here. In addition, although taxonomic and phylogenetic beta diversity co-varies, comparison of distance-decay slopes to previous work [23,24,70] suggests that these metrics are not redundant and likely provide complementary information [46]. Patterns found here specifically suggest that—relative to other bacterial systems—increasing spatial distances lead to relatively modest increases in the degree of taxonomic turnover but relatively large increases in phylogenetic turnover.

Upon observing significant vertical structure we evaluated whether the vertical patterns of the sampled bacterial communities were governed primarily by deterministic or stochastic processes. Analyses of phylogenetic structure can complement analyses of taxonomic structure, potentially providing additional insights into the factors that shape local communities [5,47]. Our correlation analyses indicated that phylogenetic structure was strongly correlated with the measured environmental variables. This observation is consistent with the significant mean value of NTI across all bacterial communities, highlighting the importance of deterministic ecological selection in driving the community assembly of bacteria. This also agrees with several previous studies in a wide range of environments [11,71,72], which showed that microbial communities had a tendency to be more phylogenetically clustered than expected by chance. Observed phylogenetic clustering in bacterial communities could also be the result of biotic interactions (*e*.*g*. facilitation and competitive exclusion) [73] as well as ecological diversification of closely related species [74]. However, given the significant mean NTI value and the strong correlation between the environmental variables and phylogenetic structure, it is unlikely that these processes primarily influence the bacterial communities in our system.

On the other hand, we observed that 5 of 16 bacterial communities were phylogenetically random, which suggested that the bacterial community assembly in these soil horizons of the permafrost core was mainly determined by stochastic processes. According to neutral theory [7], these results imply that random but spatially limited dispersal could be occurring. Previous studies have also emphasized the role of historical factors in the assembly of microbial communities, and revealed that spatial patterns of microorganisms can be attributed to the effects of historical factors [6,20]. This is especially true in our permafrost system given that historical colonization events may influence the present-day bacterial composition and distribution patterns. That is, the sequence information obtained in this study would be derived primarily from dead or inactive cells, many of which may have been deposited before the soil was perennially frozen, although a low level of bacterial activity may also exist [27,45]. Furthermore, the past depositional environments may also have an effect on the present-day bacterial communities, because the Qinghai-Tibet Plateau is characterized by the complex geological evolution processes, and formed by land uplift in the order of 3000 m over the past 2 million years [75]. In general, our results confirm previous findings [6,8–10,24], and indicate that stochastic and deterministic processes are together responsible for the assembly of permafrost bacterial communities. In our case, however, the analysis of variation partitioning showed a stronger influence of environmental component on the bacterial community composition, suggesting that the vertical structure of bacterial communities studied here was governed primarily by deterministic ecological selection imposed by physicochemical environmental conditions. Similar results were also found using partial regression analysis, in which partial correlation coefficients for both taxonomic and phylogenetic beta diversity metrics were significant for environmental distance but not for spatial distance.

Although the main aim of this study was not to examine the specific environmental factors that determine the biodiversity patterns, our results do suggest that they show potential in affecting the bacterial alpha diversity, phylogenetic structure, and spatial turnover across the permafrost core. Multiple stepwise regression analysis revealed that soil conductivity and pH were the most important explanatory variables for bacterial alpha diversity and phylogenetic structure, respectively. The change in taxonomic and phylogenetic community composition was significantly correlated with most measured soil variables, especially the soil conductivity and organic carbon content. These results accord with many previous findings, emphasizing the importance of pH in explaining phylogenetic structures of lacustrine bacterioplankton communities [76], as well as electrical conductivity salinity [77] and soil carbon content [60] in structuring microbial communities. Other factors, such as extreme temperature, low water activity and background radiation in permafrost system, may cause environmental stresses for indigenous microorganisms, and in part contribute to the deterministic assembly process observed in this study. Moreover, seasonal freeze–thaw cycles prevailing in active layer of permafrost core may have a direct connection with soil physicochemical disturbances [78], and will also be a major factor that imposes deterministic influence on the bacterial communities. This inference is supported by a previous finding suggesting that ecosystem disturbances can result in community assembly of closely related crustacean zooplankton species in freshwater environments [79], and is in agreement with a report that environmental instability leads to the prevalence of significant phylogenetic clustering in bacterial communities [80].

In conclusion, our study reveals the vertically-structured patterns of bacterial communities and infers the ecological processes driving these patterns in a permafrost core. We observed that the bacterial communities were not randomly distributed along the soil core, but rather showed a vertical distance-decay relationship. The vertical distribution of bacterial communities was mainly driven by deterministic processes such that the observed distance-decay relationship was most likely the result of physicochemical environmental conditions (primarily soil pH, conductivity, and organic carbon content), although stochastic processes were also involved. Our findings highlight the importance of considering information on both the taxonomic and phylogenetic structure of microbial communities and of using multiple lines of evidence to carefully evaluate whether distance-decay relationships indicate a strong influence of deterministic or stochastic processes. Our study further contributes to the emerging body of literature aimed at understanding the microbial ecology of permafrost [27], a key reservoir of soil carbon stocks.

## Supporting Information

S1 Fig Neighbor-joining phylogenetic tree inferred from representative 16S rRNA gene sequences of permafrost bacterial phylotypes. Bootstrap values above 50% are shown as a percentage of 1000 replicates. The scale represents the number of mutations per nucleotide position. Numbers in parenthesis represent the number of phylotypes and orders assigned to each division respectively.
(TIF)

S2 Fig The relationships of taxonomic dissimilarity (a and b) and betaMNTD (c and d) versus vertical distance and environmental heterogeneity (environmental distance), respectively. Solid lines represent linear regressions and the significance levels are determined by Mantel test (9999 permutations).
(TIF)
