# Supplementary figures and images for: Relative Roles of Deterministic and Stochastic Processes in Driving the Vertical Distribution of Bacterial Communities in a Permafrost Core from the Qinghai-Tibet Plateau, China
Source: PLoS One. 2015 Dec 23;10(12):e0145747. doi: 10.1371/journal.pone.0145747 (PMC4689587; doi:10.1371/journal.pone.0145747)

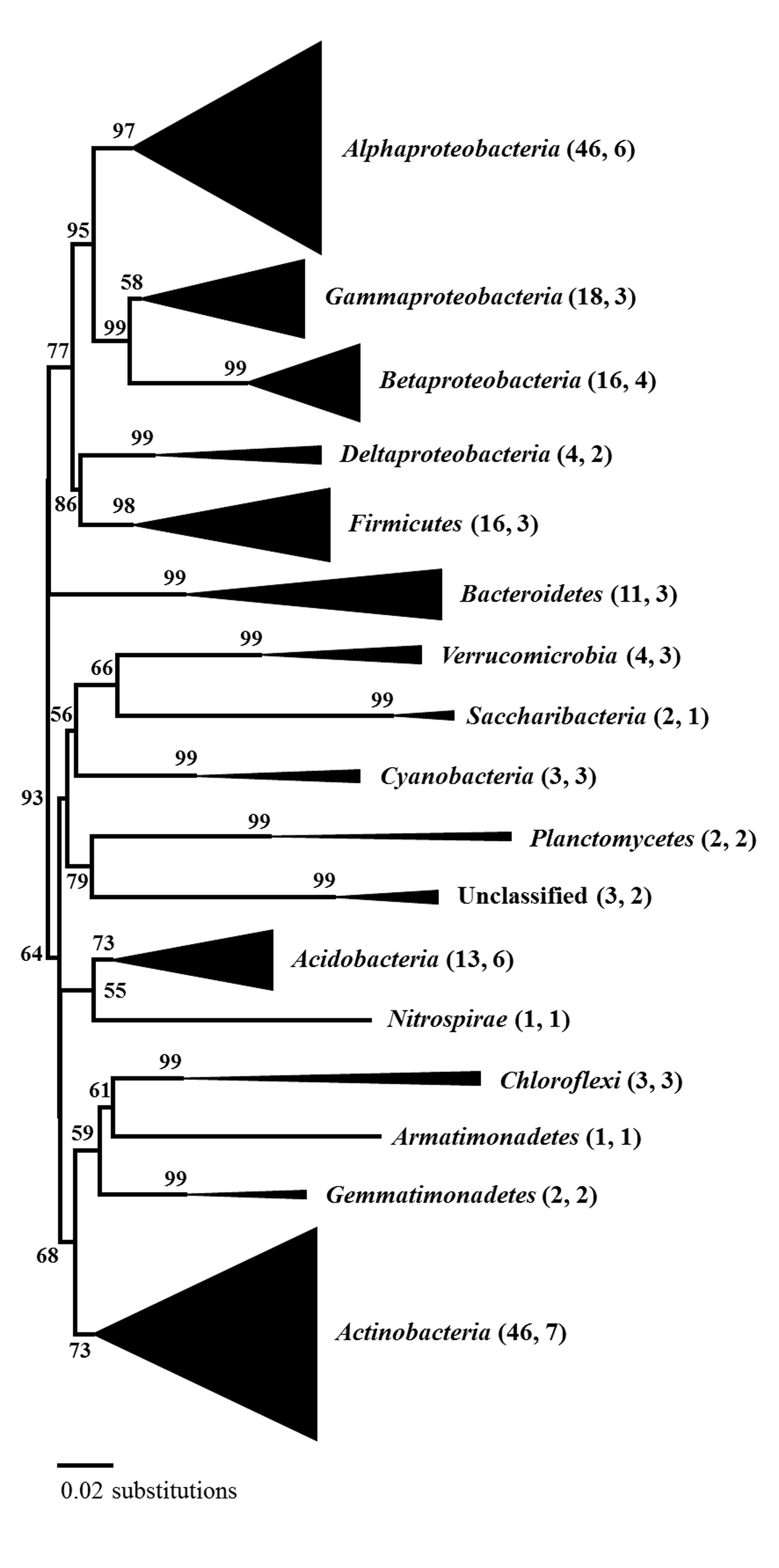

Supplement: S1 Fig — Bootstrap values above 50% are shown as a percentage of 1000 replicates. The scale represents the number of mutations per nucleotide position. Numbers in parenthesis represent the number of phylotypes and orders assigned to each division respectively. (TIF) [file pone.0145747.s001.tif]

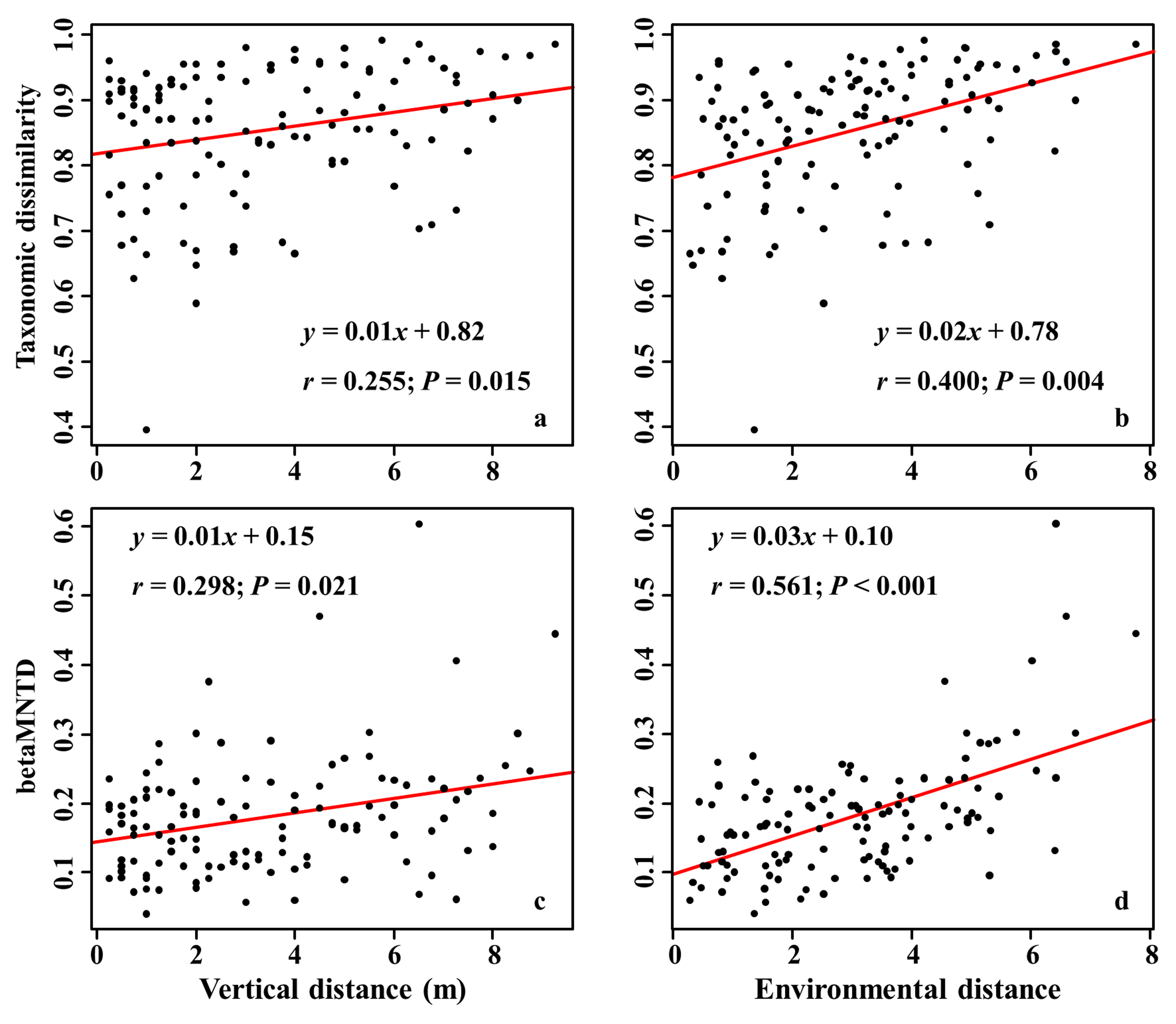

Supplement: S2 Fig — Solid lines represent linear regressions and the significance levels are determined by Mantel test (9999 permutations). (TIF) [file pone.0145747.s002.tif]
